# Convolutional Neural Networks for Spectroscopic Analysis in Retinal Oximetry

**DOI:** 10.1038/s41598-019-47621-7

**Published:** 2019-08-06

**Authors:** Damon T. DePaoli, Prudencio Tossou, Martin Parent, Dominic Sauvageau, Daniel C. Côté

**Affiliations:** 1 Université Laval, CERVO Brain Research Center, Neuroscience, Quebec City, Québec, G1V 0A6 Canada; 2 Université Laval, Center for optics, photonics and lasers (COPL), Physics Engineering, Quebec City, Québec, G1V 0A6 Canada; 3 Université Laval, Department of software engineering and informatics, Quebec city, Québec, G1V 0A6 Canada; 4 Zilia inc., Quebec City, Québec, G1V 0A6 Canada; 5 University of Alberta, Chemical and Materials Engineering, Edmonton, Alberta T6G 1H9 Canada

## Abstract

Retinal oximetry is a non-invasive technique to investigate the hemodynamics, vasculature and health of the eye. Current techniques for retinal oximetry have been plagued by quantitatively inconsistent measurements and this has greatly limited their adoption in clinical environments. To become clinically relevant oximetry measurements must become reliable and reproducible across studies and locations. To this end, we have developed a convolutional neural network algorithm for multi-wavelength oximetry, showing a greatly improved calculation performance in comparison to previously reported techniques. The algorithm is calibration free, performs sensing of the four main hemoglobin conformations with no prior knowledge of their characteristic absorption spectra and, due to the convolution-based calculation, is invariable to spectral shifting. We show, herein, the dramatic performance improvements in using this algorithm to deduce effective oxygenation (SO_2_), as well as the added functionality to accurately measure fractional oxygenation (${{\bf{SO}}}_{{\bf{2}}}^{{\boldsymbol{f}}{\boldsymbol{r}}}$). Furthermore, this report compares, for the first time, the relative performance of several previously reported multi-wavelength oximetry algorithms in the face of controlled spectral variations. The improved ability of the algorithm to accurately and independently measure hemoglobin concentrations offers a high potential tool for disease diagnosis and monitoring when applied to retinal spectroscopy.

## Introduction

Retinal oximetry is a non-invasive technology drawing considerable attention in the medical field due to its ability to give unprecedented information on the vasculature health of the eye. There is considerable evidence that malfunction of the vasculature on the retina can result in, or be an indication of, serious eye diseases such as diabetic retinopathy (DR)^1–8^, retinal vessel occlusions^9–13^, glaucoma^12,14–18^, retinitis pigmentosa^19,20^, retinopathy of prematurity^21^ and age related macular degeneration (AMD)^22^. Recently, retinal oximetry has even shown the possibility of non-invasively monitoring some neurodegenerative diseases^10^. This being said, the abundance of research performed using retinal oximeters has not yet translated into full deployment in clinical settings; mostly due to the semi-quantitative nature of measurements caused by the complex optical properties of the biological tissues on the retina^23–27^.

Retinal oximeters typically rely on either two, three or multi-wavelength analysis. Few-wavelength (<4) techniques have a long history and have been improved on greatly over the years^28–33^, however, the general method is similar. Briefly, few-wavelength techniques require images acquired on and off isosbestic wavelengths for oxygenated and deoxygenated hemoglobin and a user-calibrated optical density ratio (ODR) method to provide oximetry measurements on large blood vessels^34–36^. While the technique has flourished in academia, in recent years the measurement integrity of the technique has been questioned in terms of quantitative reproducibility. Specifically, commercially available two-wavelength imaging oximeters have displayed inconsistent oxygenation measurements caused by blood vessel sizes^35^, scattering and cataract variations^37,38^ and flash intensities^39^. Monte Carlo simulations on the error inherent to two-wavelength retinal oximetry has also shown the importance of proper calibration^40^ as well as the possible errors cause by vessel diameter and melanin concentration in the retinal epithelium^41^. Furthermore, many disease studies draw similar conclusions, but with different quantities. For example, in the progression of diabetic retinopathy (Table 1), the leading cause of blindness in adults^3,4,6,7,42,43^.

**Table 1:** Summary of retinal oximetry investigation of diabetic patients with or without diabetic retinopathy.

Source	Vasculature	Control	No DR	NPDR	PDR
Hammer *et al*.^4^	SO2−A	97 ± 4	—	100 ± 5	101 ± 4
Imedos	SO2−V	63 ± 5	—	75 ± 5	75 ± 8
Hammer *et al*.^42^	SO2−A	97 ± 6	—	97 ± 6	—
Imedos	SO2−V	66 ± 5	—	68.8 ± 7	—
Hardarson *et al*.^7^	SO2−A	93 ± 4	—	—	100 ± 5
Oxymap T1	SO2−V	58 ± 6	—	—	67 ± 8
Khoobehi *et al*. (2013)	SO2−A	92 ± 4	96 ± 9	102 ± 10	104 ± 9
Oxymap T1	SO2−V	57 ± 6	59 ± 8	67 ± 8	67 ± 10
Jorgensen *et al*.^3^	SO2−A	95 ± 1	94 ± 2	96 ± 1	99 ± 2
Oxymap T1	SO2−V	63 ± 1	64 ± 2	66 ± 2	68 ± 2
Guduru *et al*.^6^	SO2−A	91 ± 4	89 ± 8	96 ± 14	100 ± 7
Oxymap P2	SO2−V	53 ± 6	53 ± 10	63 ± 13	66 ± 11

The source column describes the source article and the machine used in the measurement. (Abbreviations: SO_2_ − A = Arterial oxygenation; SO_2_ − V = Venous oxygenation; No DR = diabetic without retinopathy; NPDR = non-proliferative diabetic retinopathy; PDR = proliferative diabetes).

With this knowledge, it is clear that systemic improvements must be made in retinal oximetery if it is to become a diagnostic device capable of being integrated into a clinical workflow. To achieve more accurate oximetry measurements and to make calibration-free systems, one solution is to include more wavelengths in the analysis to allow automatic compensation of wavelength-dependent scattering and absorption. To this end, a similar, calibration-free, solution to the ODR method used in two-wavelength oximeters was proposed by Hammer *et al*. using a 4-wavelength approach which normalizes the spectra to more than one hemoglobin isosbestic point^44^. Techniques that detect greater than 4 wavelengths routinely deploy a statistical regression method to fit the known absorption profiles of oxygenated and deoxygenated hemoglobin. Since the signal to noise ratio is proportional to the square root of the number of wavelengths considered, the more wavelengths measured, the more robust the oximetry measurement can be^45^. Furthermore, not only do these techniques allow for more robust measurements with respect to noise, they also improve the separation of hemoglobin absorption from parasitic optical attenuations such as tissue scattering, glint, and absorption from the other chromophores present in the eye. These methods, however, are not without their own flaws and, as will be seen in the present work, can be further improved upon using modern neural network approaches to achieve a precision of oximetry required for clinical use.

Firstly, as with two-wavelength techniques but to a lesser degree, multi-wavelength algorithms are sensitive to the wavelength-dependent variability from the different absorbers and scatterers in the eye. Simply-put, this is due to the optimization problem in regression analysis being a minimization of the error between the measured spectra and its respective fit using the regression components provided. This can become a problem when the best fit does not directly give the most accurate calculation of oxygenated hemoglobin, namely in the cases where the regression analysis either is missing components that are contributing to the spectral shape, or has too many components allowing untrue solving options.

Secondly, spectral mis-calibration is a problem that can appear in spectroscopy applications and often goes unnoticed. This occurs in oximetry when a measured spectra for a known chromophore and the reference spectra used to fit said chromophore is not wavelength-matched. This can play a pronounced role in oximetry techniques that use isosbestic points for calibration (2–4 wavelength techniques) as even highly-cited sources of reference absorption spectra for hemoglobin can vary; this was shown recently, in-depth, in near infrared (NIR) oximetry^46–49^. Adding this to possible spectrometer mis-calibration and spectral temperature variations, spectral shifting can be an important variable in measurement precision and accuracy.

Thirdly, retinal oximetry has historically focused on effective oxygen saturation (SO_2_). This is by definition the ratio of hemoglobin carrying oxygen to hemoglobin capable of carrying oxygen (oxygenated hemoglobin (HbO_2_) and deoxygenated hemoglobin (Hb))^50^. The mathematical representation of oxygen saturation (SO_2_) can be found in equation 1, where ${{\rm{C}}}_{{{\rm{HbO}}}_{2}}$ is the concentration of oxygenated hemoglobin and C_Hb_ is the concentration of deoxygenated hemoglobin. This is a useful parameter since it describes the oxygen carrying capacity, however, as it does not take into account dyshemoglobins (hemoglobin conformations which cannot carry oxygen), it does not directly measure the amount of circulating oxygen. A direct measurement of the oxygen concentration is only available in the calculation of fractional oxygen saturation (${{\rm{SO}}}_{2}^{fr}$) as it includes the most commonly present dyshemoglobins in humans: carboxyhemoglobin (COHb) and methemoglobin (MeHb), each making up roughly 1% of hemoglobin in healthy individuals^50^. ${{\rm{SO}}}_{2}^{fr}$ can be seen in equation 2, with the newly represented concentrations of COHb and MeHb, C_COHb_ and C_MeHb_, respectively^51^. Contrary to their traditional exclusion in retinal oximeters, knowing and accounting for these conformations is critical as their concentrations are dynamic and can be a source of considerable error, as has been shown in pulse oximetry^52–54^. COHb levels vary greatly between individuals, with the main factor of variation being frequency of smoking and living environment air pollution^55–58^. Healthy individuals that do not smoke have an average C_COHb_ of approximately 1%; however, due to environmental differences and working conditions, this can rise to as high as 3%^58^. Cigarette smokers in particular can have highly elevated COHb levels (5–15%), depending on smoking habits and time since last smoke inhalation^55–57^. Furthermore, levels can reach upwards of 40% in cases of carbon monoxide poisoning^59^. MeHb is also, on average, approximately 1% in healthy individuals^51^. However, during methemoglobinemia conditions (defined as C_MeHb_ > 2%) the concentration can increase to nearly 70% before death, and is more common than expected^60^. Moreover, below a fractional concentration of 15% of either dyshemoglobin, there are no observable symptoms, which further shows the need for oximetry techniques to be able to sense and account for their presence automatically.

1 $${{\rm{SO}}}_{2}=\frac{{{\rm{C}}}_{{{\rm{HbO}}}_{2}}}{{{\rm{C}}}_{{\rm{Hb}}}+{{\rm{C}}}_{{{\rm{HbO}}}_{2}}}$$

2 $${{\rm{SO}}}_{2}^{fr}=\frac{{{\rm{C}}}_{{{\rm{HbO}}}_{2}}}{{{\rm{C}}}_{{\rm{Hb}}}+{{\rm{C}}}_{{{\rm{HbO}}}_{2}}+{{\rm{C}}}_{{\rm{COHb}}}+{{\rm{C}}}_{{\rm{MeHb}}}}$$

We present here the novel use of CNNs to overcome the aforementioned shortcomings in current retinal oximetry calculations for directly quantifying SO_2_ and ${{\rm{SO}}}_{2}^{fr}$ concurrently in a reproducible, robust way. Convolutional neural networks (CNNs) have been finding more and more use in the biomedical field both in image recognition^61–66^ and spectral identification^67^. For spectroscopy, CNNs have incredible advantage over statistical regression techniques, owed mainly to their ability to learn and weigh the importance of different spectral regions automatically. Furthermore, they learn this weighting of spectral characteristics with no prior knowledge of the constituent’s absorption spectra. This is extremely interesting for heterogeneous tissue spectroscopy where the total attenuation is measured, as the individual attenuation coefficients for each unique structure in the optical integration volume are not always well defined, and in this case need not be. The challenge with using CNNs in retinal oximetry is to obtain large enough datasets with realistic optical variations but, most critically, with validated oxygenation measures. This difficulty results from the lack of gold-standard measurements in tissue oximetry of the eye, the prohibitive costs associated with patient measurements if there was such a technique to provide valid training targets, and the fact that current optical phantoms are too simplistic to replicate the eye appropriately.

Our solution is to train a CNN solely on a large number of simulated diffuse reflectance spectra which accurately mimic the variability in *in vivo* measurements on the optic nerve head (ONH) in humans. The CNNs were compared with several reported algorithms in an un-biased way on a wide range of test data-sets to show it’s non-incremental improvements in oximetry calculation performance. This technique of training using simulated spectra based on only a few *in vivo* measurements allows us to have an abundance of training data not traditionally available in biomedical applications. In an attempt to validate that this training technique could indeed create a CNN that had improved accuracy for *in vivo* measurements, where there are many unknowns and much optical property variation, we compared the performance of the CNN algorithms on spectra having variable contributions from chromophores not seen in the training data to regression algorithms that did not have the chromophores available for fitting in their analysis.

## Methods

### Data Creation

**Figure 1:**
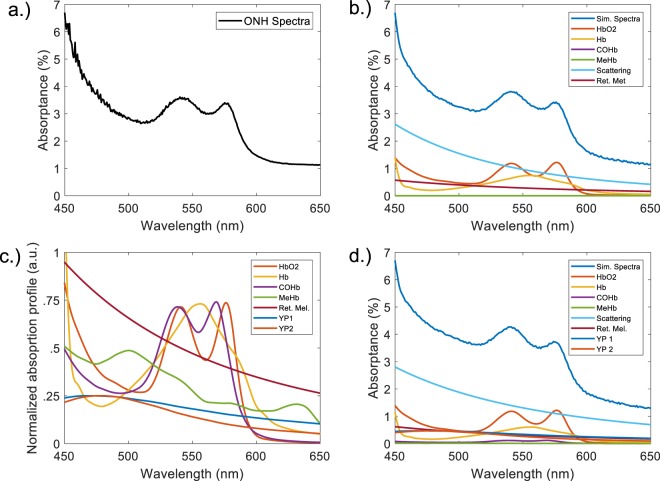
Data creation. (**a**) Example spectrum taken *in vivo* on a human optic nerve head with an SO_2_ of 68% (**b**) Randomly simulated spectrum with an SO_2_ of 68%. Components are plotted to scale of their randomly generated amplitudes for this specific spectra. (**c**) Normalized absorption coefficient spectra used in simulated spectra creation. (**d**) Randomly simulated spectra with an ${{\rm{SO}}}_{2}^{fr}$ of 68%, COHb present at a 6% fraction and a random combination of yellow protein contributions. Components are plotted to scale of their randomly generated amplitudes for the specific spectrum. Abbreviations: ONH = Optic nerve head; Ret. Mel. = Retinal Melanin; YP1 = Yellow protein 1; YP2 = Yellow protein 2.

The method focuses on demonstrating the superiority of neural networks to extract oxygenation values from simulated data. The models used to simulate the data are based on our experience with *in vivo* measurements. Specifically, *in vivo* spectra previously acquired from the ONH of subjects using a multi-wavelength oximetry device (Zilia Inc., Quebec City, Canada) were used to model the simulated datasets based on the absorption coefficients of visible-wavelength chromophores present in the eye. An example of an experimentally measured spectra from a human ONH can be found in Fig. 1(a). The 100 *μ*m spot size allows us to ignore chromatic aberrations often observed in high resolution scanning systems. To model the highly scattering myelinated structure of the ONH we added a wavelength-dependent scattering component to account for optical path-length variations^68,69^, as has been done routinely in the past for retinal oximetry applications^18,45,70,71^. Specifically, the normalized scattering factor proposed by Jacques *et al*. was applied with variable terms used for the reduced scattering coefficient and the scattering power^72^. We did not consider contributions coming from choroidal-vasculature cross-talk as it has been shown that minor contributions occur below 600 nm^40^. Absorption coefficients for the following chromophores were included in the data creation: HbO_2_, Hb, COHb, MeHb^73^ and retinal melanin^74^. A variable-magnitude constant factor (CF) was added to account for changes in light intensity. The mathematical representation of the data creation for a simulated spectra (S1) is shown in equation 3. A variable blood volume factor (B) was multiplied to the hemoglobin absorption component to vary the overall hemoglobin absorption. The variable combination of reflected intensity, wavelength-dependent scattering and reflections, as well as variable blood volumes can provide a reasonable simulation of different blood vessel sizes and varying amounts of specular reflection (also known as glint). The result of all these factors created extremely variable datasets, as one would expect to find in real life situations due to the vast variability in human eyes. The addition of all the fractional hemoglobins (including dyshemoglobins) in a given spectra were constraint to always equal to 100%. The known concentration for each absorber was saved in a separate file and either used for training (only in the case of CNN) or evaluation.

3 $$\begin{array}{rcl}{\rm{S}}1 & = & ({{\rm{C}}}_{1}\ast {\mu }_{{\rm{a}},{{\rm{HbO}}}_{2}}+{{\rm{C}}}_{2}\ast {\mu }_{{\rm{a}},{\rm{Hb}}}+{{\rm{C}}}_{3}\ast {\mu }_{{\rm{a}},{\rm{COHb}}}+{{\rm{C}}}_{4}\ast {\mu }_{{\rm{a}},{\rm{MeHb}}})\ast {\rm{B}}\\  &  & +{{\rm{C}}}_{5}\ast {\mu }_{{\rm{a}},{\rm{Retinal}}{\rm{Melanin}}}+a\ast {(\frac{\lambda }{500{\rm{nm}}})}^{-b}+{\rm{CF}}\end{array}$$

After analyzing performances of the extraction algorithms on spectra made with the known chromophores, we created a subset of spectra (S2) having two variable yellow-lens-protein contributions added, as defined by Dillon *et al*.^75^. This addition was chosen as it is well established that there is a presence of yellowing proteins on the lens of aged and diseased individuals and therefore it is often in the optical path of the spectroscopic detection^38,75,76^. The absorption spectra for the yellowing proteins were purposely left out of the linear regression analyses and a CNN was trained on data without their inclusion. This constituted an integral performance comparison as there are often unknown contributions in a detected spectra in *in vivo* tissue spectroscopy. CNNs require no previous knowledge of reference spectra and therefore can learn to be completely invariant to this addition with *in vivo* training data and so we also included another CNN, trained on data that had yellow protein contributions included, to show this effect. The first CNN without yellow proteins in the training data exemplifies how much more robust the machine-learned weighting is when presented with unknowns; the second CNN, with yellow proteins in the training data, how improved a CNN can become with *in vivo* training. The mathematical representation of the data creation for a simulated spectra (S2) is shown in equation 4.

4 $${\rm{S}}2={\rm{S}}1+{{\rm{C}}}_{7}\ast {\mu }_{{\rm{a}},{\rm{Yellow}}{{\rm{Protein}}}_{1}}+{C}_{6}\ast {\mu }_{{\rm{a}},{\rm{Yellow}}{{\rm{Protein}}}_{2}}$$

The ranges of coefficients used in the elaboration of the spectra, defined in equations 3 and 4, are shown in Table 2. Due to the focus on spectra originating from the ONH, the melanin contribution was assumed to be quite small and this matched the experimental reference spectra. A normalized representation of the chromophores’ absorption coefficients can be found in Fig. 1(c). For comparison, an *in vivo* spectral measurement taken on a patient’s ONH with a predicted ${{\rm{SO}}}_{2}^{fr}$ of 68% is shown in Fig. 1(c) along with randomly simulated spectra with ${{\rm{SO}}}_{2}^{fr}$ = 68% for both a typical non-smoker (COHb and MeHb <= 1%) and a typical smoker with an elevated COHb concentration of 6% in Fig. 1(b,d), respectively.!

**Table 2:** Variable ranges for spectral components.

Variable	B	C1 (%)	C2 (%)	C3 (%)	C4 (%)	C5,C6,C7	a	b	CF
Range	[−0.1–0.1]	[0–100]	[0–100]	[0–100]	[0–100]	[0.01–0.05]	[0–3]	[0.1–3.0]	[0–1]

### Algorithms

#### Convolutional Neural Networks

Opthalmology is a field that, in recent years, has seen a considerable increase in the implementation of machine learning (ML) due to impressive advancements in deep learning architectures and novel optical technologies providing more information from the retina. Recently, ML has been exploited to analyze visible images of the retina for DR diagnosis, OCT datasets to monitor retinal changes in AMD and even to automatically segment individual photoreceptors in adaptive optics ophthalmoscopy^77–79^. While others have focused on imaging applications, we present the use of ML to analyze 1-dimensional (1D) spectroscopic information.

Of the many state-of-the-art machine learning algorithms attempted, CNNs achieved the best performance of oximetry calculation as it is by nature optimally suited for handling spectral signals. A full description of CNNs is beyond the scope of this work (see the references^80–83^ for a comprehensive overview), but this section will describe them briefly. CNNs can consist of several layers of operations which transform the input into a desired output, in our case, the input is a spectrum with many optical contributions and the output is the four hemoglobin quantities. While the CNN has no previous knowledge of any hemoglobin reference spectra, its training allows it to perform calibration-free calculations on their concentrations.

The training process is governed by two main operations: the convolution and the pooling. A convolution is the integral of point-wise multiplications between network parameters and an input. These parameters are random at the beginning of the optimization and become specific to the task at hand as the network is introduced to more data. These learned parameters are termed filters.

Simply put, in our case, the convolution operation can be seen as a scalar product between multiple patches of the spectra and multiple filters. Moreover, many different filters are applied to the same spectra in order to extract different types of features. The set of filters that are chosen creates what is called a kernel and all the features extracted are designated as “feature maps” (each map is associated to a filter). The intelligence of the algorithm stems from the fact that the weights of all the filters are learned from the data itself by optimization, thus only the data dictates which features are important and should be extracted from the spectra. This is very important, for instance, if the data is noisy, the network will learn filters that are resilient to noise.

Subsequently the pooling step is applied to make the extracted features invariant to their position. Explicitly the feature maps are passed through a type of non-linear downsampling function allowing a pre-defined variable to be considered over all the features (in our case, we take the max value as our variable, therefore we perform “max-pooling”). The explanation of this step is that the exact location (wavelength) of a feature (*i.e*. the HbO_2_ peak around 580 nm) is less important than its relative location to other features within a certain range, which effectively gives the algorithm its prediction invariance to spectral shifting in this range.

Using the Keras API in Python, we trained four CNN algorithms in total: (1.) a short wavelength range (500–600 nm) network trained without yellow protein absorption in the spectra (CNN-SW), (2.) a long wavelength range (450–650 nm) network trained without yellow protein absorption spectra (CNN-LW), (3.) a short wavelength range (500–600 nm) network trained with yellow protein spectra included (CNNYP-SW), (4.) a long wavelength range (450–650 nm) network trained with yellow protein spectra included (CNNYP-LW).

The adopted network has a feature extractor consisting of CNN layers and a multi-output regressor which is a fully connected layer having the four hemoglobin concentrations as outputs. The amounts of SO_2_ and ${{\rm{SO}}}_{2}^{fr}$ were then calculated using equations (1) and (2) in a similar fashion to post-linear regression analysis. The four networks all have the same architecture, only the training data and wavelength range is varied. The feature extractors have three convolution layers having respectively 128, 128, and 64 filters. Each convolution layer has a kernel size of 25 and is followed by an exponential linear unit (ELU) activation and max pooling layer with a pooling size of 2. The output of the feature extractor is the concatenation (or flattening) of all 64 filters of the last convolution layer which was first normalized. The choice of the ELU has two desirable properties: producing a zero-centered distribution, which can make the training faster; and having one-sided saturation which leads to better convergence. For optimization, the ADAM optimizer was used, which is essentially an adaptive version of Stochastic gradient descent. Finally, the loss function for the training optimization was the summation of the mean squared errors between the predicted values and the observed values for all four of the hemoglobin concentrations.

Specifically, the network was trained using a dataset of randomly simulated spectra as described in the Data Creation section. The set contained 20,000 simulated spectra of which 75% were used to optimize the parameters of the network and the rest to validate their performance. The validation partition was used to stop the training early when the network performance on the validation dataset was optimal, rather than optimizing on and overfitting the training dataset. While the training dataset is large, it exemplifies the major advantage of training using simulated data. Once calibration data has been acquired, the pre-trained network could be retrained in an iterative fashion with a decreased demand for experimental data. For fairness in our comparison, their is no overlap between the training set and the testing sets (used for comparison between algorithms).

#### Comparison Algorithms

We included 6 different algorithms in our comparison of the CNNs. Two of the algorithms were previously published^44,70^ and replicated in MATLAB and the other 4 were designed in-house using MATLAB. We will go over the 6 algorithms in more detail in this section.

Two-wavelength imaging oximeters require extensive calibration using measurements on and off of an artery at an isosbestic wavelength for normalizing and a non-isosbestic wavelength for contrast. The technique is sound but incomplete spectral information often leads to variable and non-quantitative oximetry measurements. To present an alternative to these techniques using only spectral data and no imaging data, the first algorithm considered was the 4-wavelength technique by Hammer *et al*.^44^. This algorithm is similar in that the oximetry calculation is based solely on a single contrast wavelength however it uses three HbO_2_/Hb isosbestic wavelengths to remove scattering and absorption variability. In this article, we will refer to this algorithm as the Hammer algorithm (HA). The pros of this algorithm are that it is fast, calibration-free, and requires only 4 wavelengths for calculation. In short, first a measured spectrum is linearly transformed such that the slope between the isosbestic points of 522 nm and 586 nm matches the reference spectra. Subsequently the data between the isosbestic points is stretched or compressed to match the 3rd HbO_2_/Hb isosbestic wavelength at 569 nm. The oxygen saturation is then calculated using the intensity at 560 nm corresponding to one of the characteristic peaks of HbO_2_. More detailed information can be found in their publication^44^. Of course, since the oxygen sensing contrast is based on a single point, this technique is susceptible to noise. Furthermore, it cannot yield measurements for ${{\rm{SO}}}_{2}^{fr}$ as it only considers the presence of HbO_2_ and Hb.

The second algorithm was a remake of a linear regression algorithm reported by Diaconu using the lsqnonneg.m function in MATLAB. In the regression analysis it included a constant component, multiple ocular media components as defined, and Hb and HbO_2_ absorption spectra^70^. Here, we will refer to the algorithm as the Diaconu algorithm (DA). This algorithm is great at *fitting* the spectra due to the many ocular media components; however, the goal in oximetry is to optimize oxygen sensing, not necessarily spectral fitting - an important distinction.

The third algorithm was a simple linear regression analysis also using lsqnonneg.m in MATLAB, but with fewer components. This regression analysis solved only using a constant factor, a fixed wavelength-dependent scattering coefficient (*a* = 1, *b* = −2, in scattering equation), retinal melanin, Hb and HbO_2_ excinction spectra^47,72–74^. This algorithm will be referred to simply as algorithm 3 (A3). A general rule for thumb in linear regression analyses are that the less components for solution, the better.

The fourth algorithm was identical to the third, with the addition of COHb and MeHb absorption spectra added to the linear regression solving components. This algorithm was, therefore, the only one that could be used to compare the performance of ${{\rm{SO}}}_{2}^{fr}$ calculation against the CNN. This algorithm will be referred to simply as algorithm 4 (A4).

As our work focuses on spectra in the visible wavelength region we examined two spectral windows for calculation in all of our in-house algorithms. The first, an intrinsically HbO_2_/Hb peak weighted range of 500–600 nm, termed small window (SW). The second, a larger range 450–650 nm, termed large window (LW) which could have advantages for instance in CNNs where weighting is learned.

### Statistical analysis

To compare the different algorithms, we looked at the mean absolute error (MAE) between the predicted value (p) and the known value (k) for SO_2_ and ${{\rm{SO}}}_{2}^{fr}$. Each test dataset included 10000 spectra and was analyzed by each algorithm. MAE was calculated using equation 5. In addition to MAE, which provides a measure of accuracy of calculations, we also looked at the standard deviation (STD; equation 6), as it provides a metric for prediction precision. The results will be presented as MAE being the highlight of the result with STD shown as the error bars.

5 $${\rm{MAE}}=\frac{{\sum }_{i=1}^{n}\,||{p}_{i}-{k}_{i}||}{n}$$

6 $${\rm{STD}}=\sqrt{\frac{1}{N}\sum _{i=1}^{N}\,{({x}_{i}-\bar{x})}^{2}}$$

## Results

### Base performance of SO_2_ calculation

**Figure 2:**
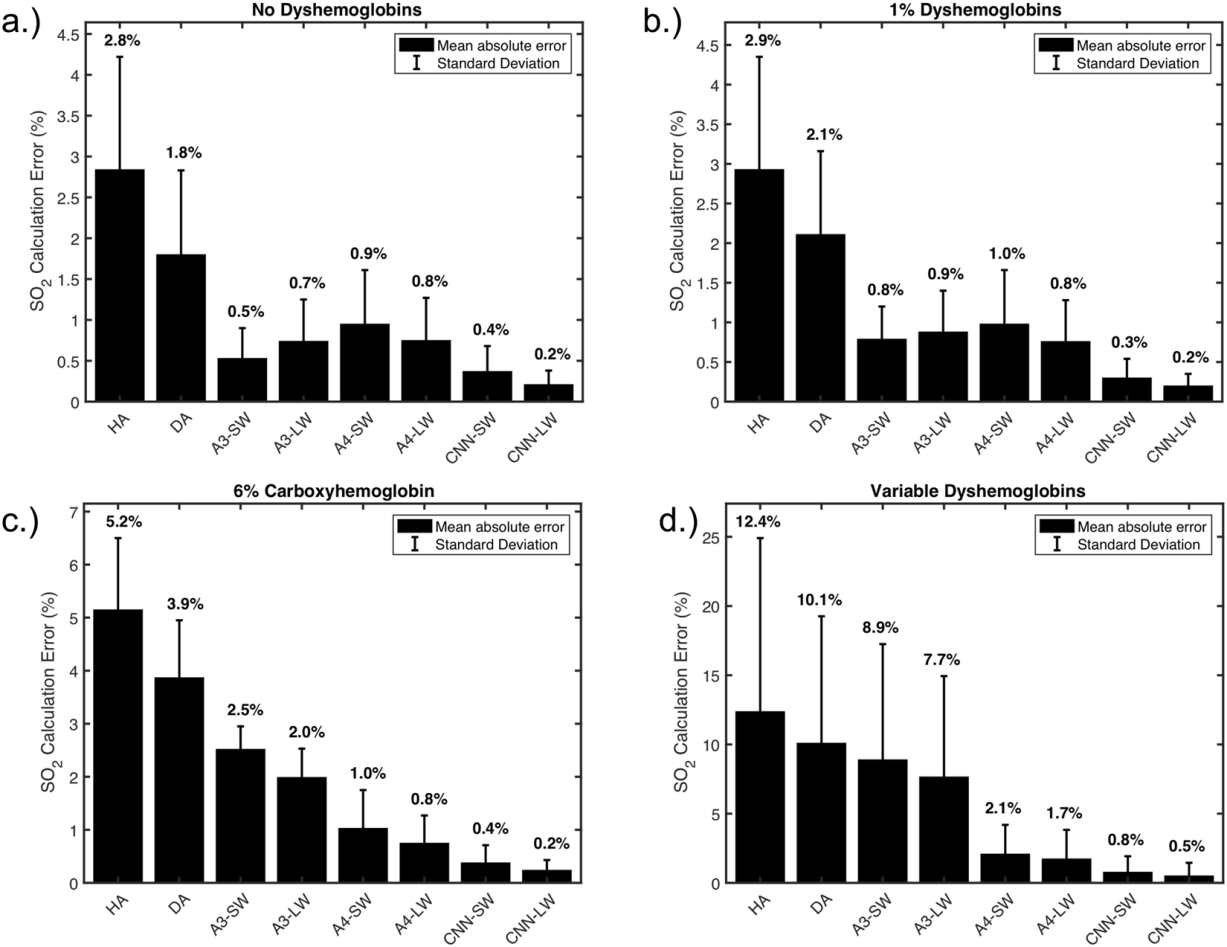
Performance of SO_2_ calculations using various oximetry algorithms. (**a**) Test dataset spectra without COHb or MeHb. (**b**) Test dataset spectra include 1% COHb and MeHb contributions to the hemoglobin absorption component. (**c**) Test dataset spectra include 6% COHb and 1% MeHb contributions to the hemoglobin absorption component. (**d**) Test dataset spectra include a random contribution of each hemoglobin conformation. The printed numbers above each bar correspond to the mean absolute error value for the given algorithm.

We first performed bench-marking of the different algorithms for the calculation of SO_2_ in traditional conditions, without the unknown yellow protein contribution. The datasets were analyzed using each algorithm and the respective SO_2_ results were compared against the known values. Four different cases were examined: (1) test spectra without any dyshemoglobins to replicate traditionally investigated scenarios, (2) test spectra having 1% of each dyshemoglobin to replicate healthy human hemoglobin concentrations, (3) test spectra having a fixed concentration of COHB of 6% and MeHb of 1% to replicate a normal smoker’s hemoglobin concentrations, and (4) test spectra consisting of completely random combinations of all the dyshemoglobins to measure general performance. In all cases both the small window and large window CNN had the least error in measuring SO_2_ as shown in Fig. 2.

### SO_2_ calculation in the presence of unknown yellow lens proteins

**Figure 3:**
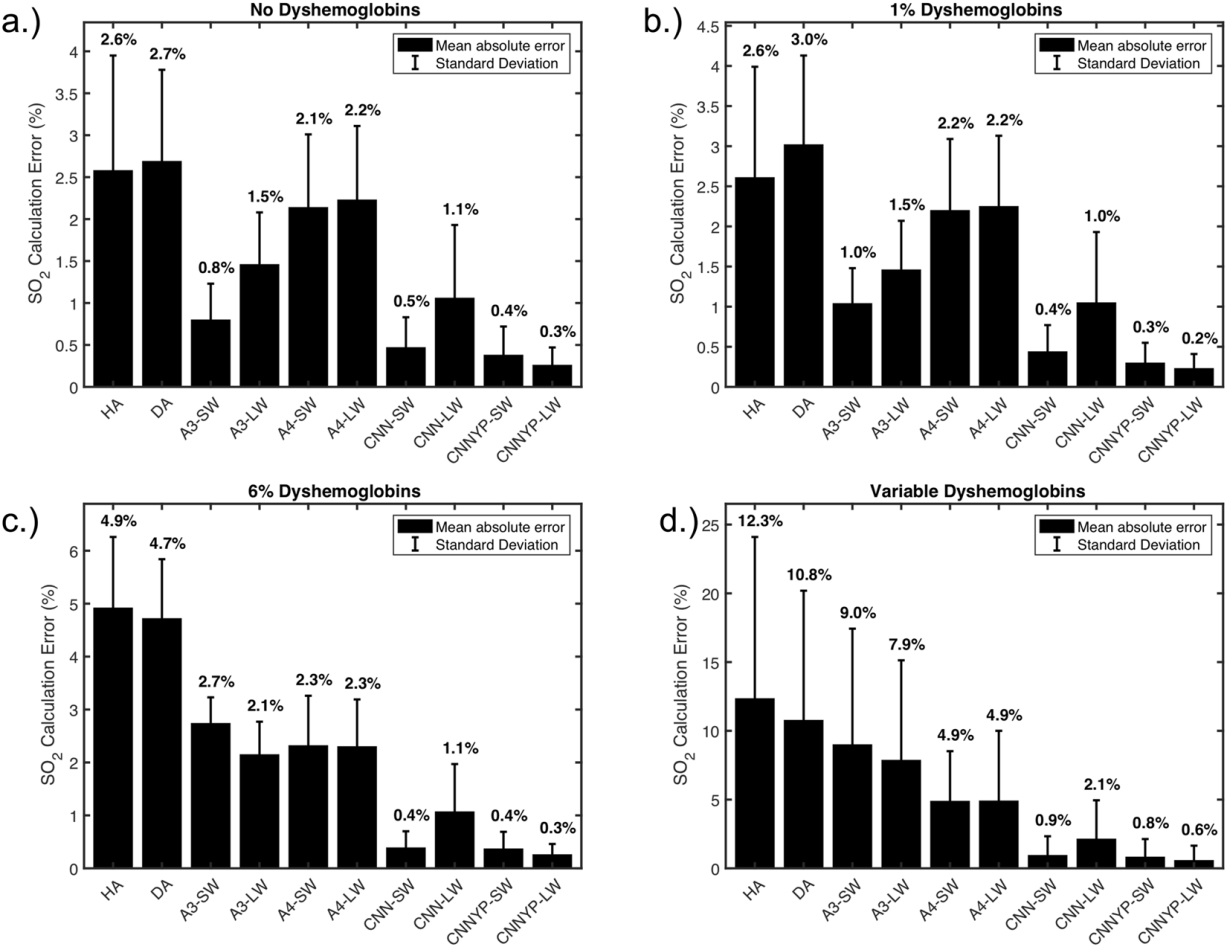
Performance of SO_2_ calculations on datasets including contributions from yellow protein components, using various oximetry algorithms. In this scenario, the linear regression analyses do not include the yellow proteins component for solving and the CNN was not trained on data having yellow proteins contributions. (**a**) Test dataset spectra without COHb or MeHb contributions. (**b**) Test dataset spectra include 1% COHb and MeHb contributions to the hemoglobin absorption component. (**c**) Test dataset spectra include 6% COHb and 1% MeHb contributions to the hemoglobin absorption component. (**d**) Test dataset spectra include a random contribution of each hemoglobin conformation. The printed numbers above each bar correspond to the mean absolute error value for the given algorithm.

In our second example we examined the robustness of the different algorithms in the presence of absorbers with unknown reference spectra. This example represents the real life scenario where some reference spectra required to perfectly fit a measured spectrum are not available or unknown. The chosen unknown absorbers in this case were two yellow proteins which are biologically relevant as they can accumulate on the lens during normal aging. For clarity, CNN-SW and CNN-LW were trained without yellow protein absorption present in the training data. We performed the same benchmarking conditions as in the previous section (no dyshemoglobins, 1% dyshemoglobins, 6% COHb and 1% MeHb, and variable dyshemoglobins) and the results are shown in Fig. 3. We also added two new algorithms to the analysis at this step, CNNYP-SW and CNNYP-LW, which are simply CNNs that have been trained using the same protocol but with training data including yellow proteins contributions.

As we see CNN-SW, having never seen the yellow proteins, still provided robust analysis in all scenarios. CNN-LW loses some performance, however, it remains on-par or better than the other non-CNN algorithms. The CNNs trained with the yellow proteins, of course, showed dramatic improvements over the other algorithms.

### Base performance of ${\bf{S}}{{\bf{O}}}_{2}^{{\boldsymbol{fr}}}$ calculation

**Figure 4:**
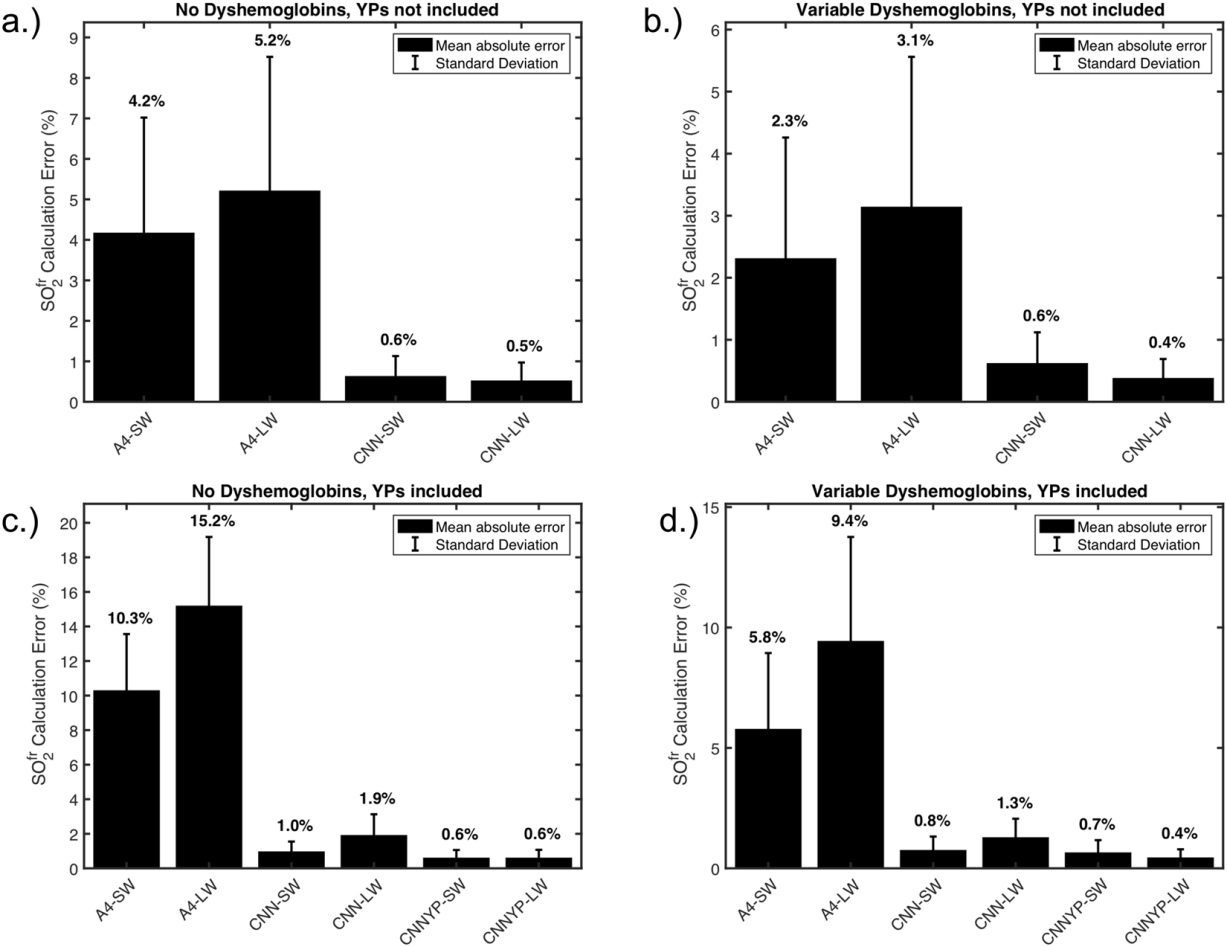
${{\rm{SO}}}_{2}^{fr}$ calculation performance on test data-sets with and without dyshemoglobins and yellow proteins. (**a**) Test data-set spectra do not include COHb, MeHb or yellow proteins. (**b**) Test data-set spectra do not include COHb or MeHb but do include a variable amount of yellow protein contributions (**c**) Test data-set spectra include random amounts of all hemoglobin conformations but do not include yellow protein contributions. (**d**) Test data-set spectra include random amounts of all hemoglobin conformations and include yellow protein contributions.

While SO_2_ can be a useful measurement, ${{\rm{SO}}}_{2}^{fr}$ may have more clinical relevance in the evaluation of hemodynamics. ${{\rm{SO}}}_{2}^{fr}$ allows the computation of the total amount of oxygen present in the blood, rather than only the ratio of oxygenated to oxygenatable hemoglobin. The measurement of ${{\rm{SO}}}_{2}^{fr}$ therefore adds a second useful variable for retinal oximetry to use towards the monitoring and diagnosis of diseases. There are two likely reasons for why ${{\rm{SO}}}_{2}^{fr}$ is routinely ignored in retinal oximetry: (1) 2-wavelength systems do not have the capability to sense it, and (2) the current algorithms used with multi-wavelength systems lack the high accuracy necessary for its calculation. As seen in Fig. 4, the CNN algorithms detect ${{\rm{SO}}}_{2}^{fr}$ with a much higher precision than the linear regression algorithms both with and without the presence of the yellow protein components. In the scenarios including yellow protein, we include the results from both CNNs to once again show the different advantages offered depending on the training.

### Noise, spectral shifting and spectral resolution resilience

**Figure 5:**
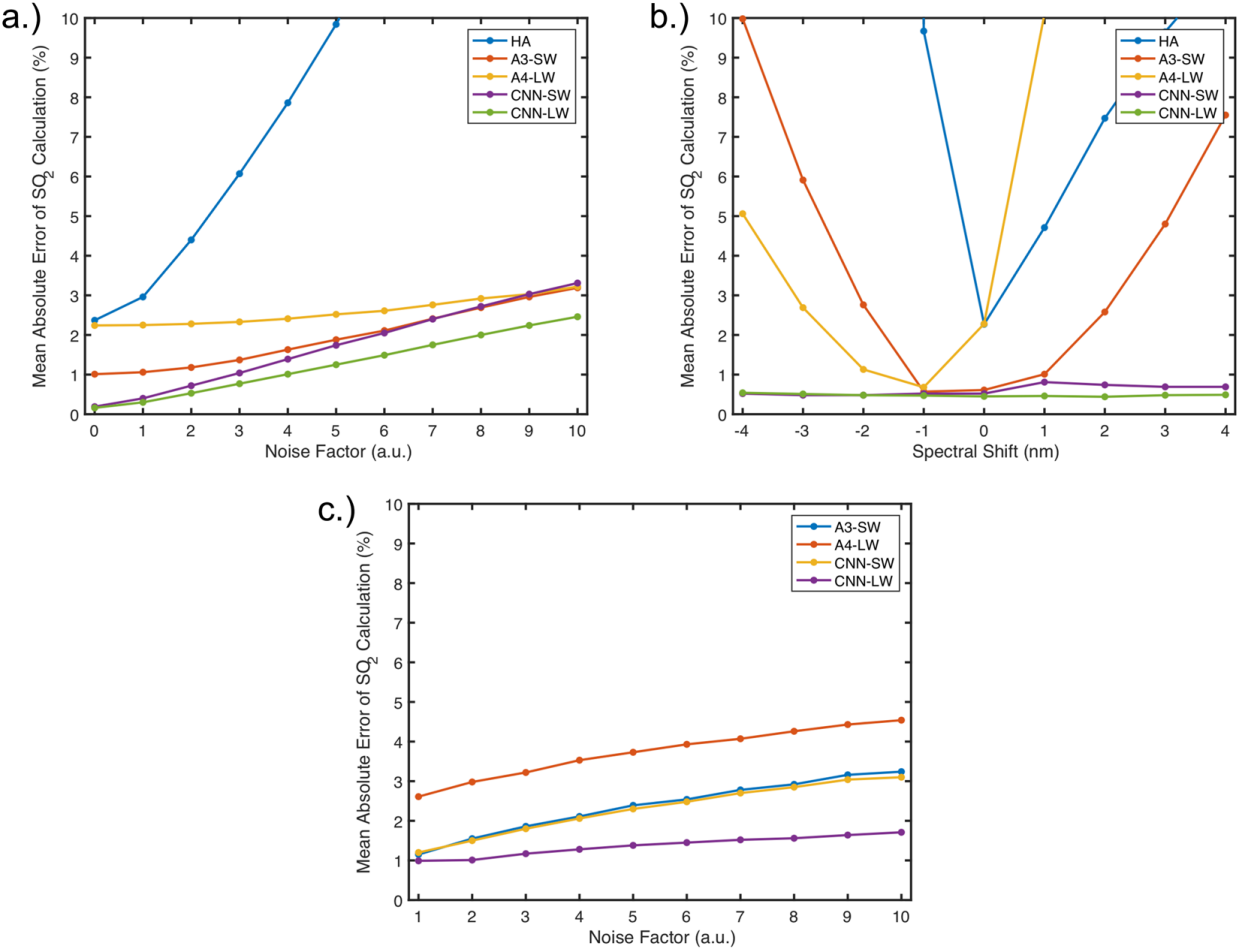
Performance of SO_2_ calculations, measured using the mean absolute error statistic, on test datasets with varying stresses. (**a**) Test datasets were created with varying amounts of noise applied to the spectra. (**b**) Test datasets were created with varying amounts of spectral shifting from reference spectra. (**c**) Test datasets were created with decreasing spectral resolution.

To analyze the resilience of the CNNs, we compared it against the top performing algorithms at calculating SO_2_ when noise, spectral shifting and lowered resolution were applied to spectra datasets having no dyshemoglobins or yellow proteins to isolate the performance variables. We also included the HA algorithm in the noise and spectral shifting analyses to exemplify the improvements even the linear regression techniques bring over few-wavelength systems. In the case of noise application, 11 test datasets were created with a fixed amount of white Gaussian noise and the algorithms were run on each dataset. As can be seen in Fig. 5a) the CNN-LW had the best performance, however the linear regression algorithms (A3 and A4) were comparable. The poor performance of the HA algorithm in this test was to be expected due to the use of a single point intensity for oxygenation measurement after isosbestic calibration.

In the case of spectral shifting between reference spectra and measurement, test data-sets were created and their corresponding wavelengths were shifted from −4 nm to +4 nm to examine the effect of spectrometer mis-calibration, temperature changes or the use of incorrect reference spectra. The HA algorithm also performed poorly in this test as the shift ultimately leads to the use of invalid isosbestic wavelengths, greatly affecting the reliability of calculations. This is the case for any two-wavelength system, and therefore a lot of attention must be placed on the system’s calibration. On the other hand, perhaps one of the most exciting advantages of using a CNN is the ability of the algorithm to be completely invariant to spectral shifting due to the convolutional nature of the calculation, as seen in Fig. 5b).

In the case of a decrease in spectral resolution, test data-sets with increasing distance between spectral points and therefore decreasing resolution were created at a fixed noise factor of 3 to apply some stress to the algorithms. Interestingly, the CNN-LW performed considerably better than CNN-SW and their statistical linear regression counterparts at SO_2_ calculation. The HA algorithm could not be included in this experiment as it uses four fixed wavelengths.

## Discussion

Our results show the improvements of CNNs, trained only using simulated data, to perform high accuracy oximetry in comparison to four-wavelength, isosbestic, algorithms and many-wavelength regression analysis techniques. We validated the CNN’s robustness in the face of unknown optical contributions by showing its ability to outperform regression analysis when analyzing spectra having simulated yellow protein absorption (commonly observed on an aged lens) unseen in the training data.

To further validate the CNN algorithm experimentally, we have applied the it to ONH spectra acquired *in vivo* and measured similar values to that of linear regression techniques, however, without known oxygenation quantities this remains a qualitative observation. To improve the certainty of the algorithm, the next step will involve measuring optical phantoms infused with oxygen-calibrated whole blood samples. Importantly, while we plan to confirm the CNN’s efficacy on these calibrated phantoms of known oxygenation, we believe that the training of the CNN using simulated data is a strength of our approach and will not likely be replaced by only experimental measurements. That said, our simulations will be refined to better resemble the experimental data if need be. Among other advantages, including experimentally-inspired simulated data allows us to: (1) greatly increase our training data and (2) teach the algorithm to ignore non-oxygen related spectral changes by adding parasitic contributions to the spectra.

While the spectra created were made to mimic those obtained from the ONH, the scaling of the approach for any retinal structure, including blood vessels, can be easily implemented. It is important to mention that traditional two-wavelength retinal oximeters can only measure oxygenation of large blood vessels due to their limited spectral information. The system we have used to base our simulations performs spectroscopy at any location on the retina (Zilia inc., Quebec City), allowing tissue oximetry of the capillary bed within the optical integration volume.

The Beer-Lambert law predicts an exponential dependence of absorbance on the photon pathlength; highly scattering media (such as the ONH, retina, blood vessels) can lead to deviations from this observation. Previously, is has been reported that ONH measurements could be better fit using a percentage-based *absorptance*, rather than taking the logarithmic form of the measurement prior to regression analysis^70^. Keeping in mind that optimizing for fit does not necessarily mean optimal oximetry calculations, this observation could be argued. However, with neural networks, the exponential form of the data does not matter, due to the fact that no knowledge on the reference absorption coefficients are needed to perform the oximetry. This represents a major advantage of CNNs in that some complicated optical properties may not need to be as thoroughly investigated if the training data is experimentally-inspired^84,85^.

The different scenarios that were tested were chosen to compare the algorithms without bias, not favoring CNNs. The first scenario we examined considered spectra without any dyshemoglobins. Even if this case is not the most biologically accurate, it was important to include it as it represents the only scenario considered by most current oximetry techniques. While CNNs always performed better, the A3-SW algorithm always should and does have the best performance of the non-CNN approaches. This can be explained by the fact that: (1) it uses the fewest solving components in its regression analysis therefore forcing the use of the HbO_2_ and Hb absorption coefficients to optimize the regression, and (2) the small wavelength window innately weights the characteristic peaks of HbO_2_ and Hb. The second case was the most biologically relevant with the concentrations of dyshemoglobins both restrained to approximately 1% - corresponding to the estimated amounts present in healthy individuals. As expected the A3 algorithm should and does lose some performance in this scenario (since they do not include dyshemoglobins in their regression analysis). In the third case, spectra represent those of a healthy individual that is also a smoker, with COHb concentrations around 6%. A4 algorithms should and do start to outperform the other non-CNN techniques at this point due to their inclusion of all hemoglobin conformations in the regression analysis. The fourth base performance test dataset consisted of a completely random combination of all the dyshemoglobins. This case showed the robustness of the various algorithms to any possible circumstance and provided a useful random metric. Overall, the first section, which included no yellow protein contributions, provided an important base performance analysis against existing techniques, in the conditions traditionally considered. Moreover, it showed the improvements CNNs can make in oximetry analysis in the presence of dyshemoglobins, making it the first technique which can accurately analyze tissue oxygenation in individuals with elevated levels of COHb and MeHb.

The second section, including the unknown yellow protein absorbers, provided a metric to compare the different algorithms when naive to the presence of a certain optical property. Specifically, this example showed two major advantages of CNNs. First, from the CNNs that are trained with no prior knowledge of the yellow proteins (CNN-SW and CNN-LW), we see that CNN-SW in particular is much more robust to the unknown addition than the regression and isosbestic analyses. We also see here that CNN-LW becomes on par in some cases with the next-best regression algorithm (A3-SW) that is naive to yellow proteins; however, it remains more robust in the the scenarios with increased dyshemoglobins. This leads us to the conclusion that if the goal is only to measure SO_2_ than the innate weighting of small wavelength range (500–600 nm) is still optimal, even for neural networks. A possible explanation is that when the neural network is trained over a large wavelength range and tested on data with which it is familiar (data without yellow protein absorption) it takes full advantage of the wavelengths that, relatively, do not say much about hemoglobin oxygenation (outside of 500–600 nm). Therefore, when a spectra has a new component (yellow protein) that is highly absorbing below 510 nm, it does not realize as readily that this is not caused by hemoglobin absorption changes. Whereas in the short wavelength range, the weights are better assigned. We also show that the improvements in using the longer wavelength range returns when the network is trained with yellow protein contributions present, allowing it to learn how to properly weight this region.

Second, from the CNNs trained on data having seen the yellow proteins (CNNYP-SW and CNNYP-LW) we better understand the importance of the fact that CNNs do not require prior knowledge of the constituents absorption spectra. This means that as long as the training data for the CNN is experimentally acquired and calibrated, it can learn to ignore non-important contributions during its optimizations, without dissecting and analyzing the each layer of the heterogenous tissue. CNNYP-SW and CNNYP-LW, we can therefore think of as the resulting accuracy we may attain if we train our network using spectra measured from calibration phantoms and *in vivo* measurements. Likewise, with the same train of thought, we could imagine including all the absorption coefficients from known (and relevant) biological chromophores found in human ocular tissue in the training data, serving only to teach the neural network what is not an important wavelength region for oximetry calculations.

The third section analyzing ${{\rm{SO}}}_{2}^{fr}$ shows that CNNs can overcome the shortcomings of classical regression algorithms in measuring dyshemoglobins by providing a drastic (relative order of magnitude) improvement in measurement accuracy. These resulting improvements can be important not only for oximetry resilience but also for diagnosing dyshemoglobin-affiliated conditions, *i.e*. carbon monoxide poisoning and methemoglobinemia. Interestingly, the regression algorithms perform better when calculating ${{\rm{SO}}}_{2}^{fr}$ on the datasets with variable amounts of each hemoglobin conformation than in the cases with no dyshemoglobins present at all (4b) vs (4a) for cases without yellow proteins contributions, and (4d) vs (4a) for cases with unknown yellow proteins contributions). This phenomenon is likely due to overestimation of the dyshemoglobins when they are present at low concentration. This is likely why most linear regression analyses choose to ignore the presence of dyshemoglobin altogether, since they are most often at low concentrations.

Overall, the examples show the strength of performance, robustness and versatility of the CNN approach to provide a new framework for the analysis of hemoglobin conformations and perhaps other biomarkers. While previous techniques vary in performance based on the scenario, the CNNs remain optimal, throughout all the sections. This is an important point, as in a device it is not desirable, nor often possible, to choose a specific algorithm to use based on the patient (ie. cataracts, smoker, methemoglobinemia). With the abundance of publications on retinal oximetry in recent years and its potential to be integrated in diagnostic efforts, we believe that switching to a more quantitative and universal system that can provide information about all hemoglobin conformations will greatly benefit oximetry analysis and applications.

## Conclusion

We have presented theoretical evidence that neural networks have great potential in retinal oximetry for the quantitative assessment of both SO_2_ and ${{\rm{SO}}}_{2}^{fr}$. Specifically, we show that the neural networks we have designed have the lowest error in the estimation of SO_2_ and that they can improve ${{\rm{SO}}}_{2}^{fr}$ prediction by roughly an order of magnitude, relative to the other oximetry algorithms considered. The additional information provided by the CNN in the form of accurate and concurrent ${{\rm{SO}}}_{2}^{fr}$ measurements could have profound effects on increasing the reliability and diagnostic applications of measuring retinal oxygenation. Furthermore, the deduction of individual dyshemoglobin concentrations may add new variables towards novel diagnostic measurements.
